# Proteomic profiling of lung immune cells reveals dysregulation of phagocytotic pathways in female-dominated molecular COPD phenotype

**DOI:** 10.1186/s12931-017-0699-2

**Published:** 2018-03-08

**Authors:** Mingxing Yang, Maxie Kohler, Tina Heyder, Helena Forsslund, Hilde K. Garberg, Reza Karimi, Johan Grunewald, Frode S. Berven, Sven Nyrén, C. Magnus Sköld, Åsa M. Wheelock

**Affiliations:** 1 Respiratory Medicine Unit, Department of Medicine Solna & Center for Molecular Medicine, Karolinska Institutet, Lung Research Lab L4:01, SE-171 76 Stockholm, Sweden; 2 Proteomics Unit (PROBE), Department of Biomedicine, University of Bergen, Bergen, Norway; 3 Department of Molecular Medicine and Surgery, Division of Radiology, Karolinska Institutet, Karolinska University Hospital, Solna, Stockholm, Sweden

**Keywords:** Chronic obstructive pulmonary disease, Bronchoalveolar lavage, Smoking, Gender difference, Proteomics, Isobaric tags for relative and absolute quantitation, Orthogonal projection to latent structure-discriminant analysis

## Abstract

**Background:**

Smoking is the main risk factor for chronic obstructive pulmonary disease (COPD). Women with COPD who smoke experienced a higher risk of hospitalization and worse decline of lung function. Yet the mechanisms of these gender-related differences in clinical presentations in COPD remain unknown. The aim of our study is to identify proteins and molecular pathways associated with COPD pathogenesis, with emphasis on elucidating molecular gender difference.

**Method:**

We employed shotgun isobaric tags for relative and absolute quantitation (iTRAQ) proteome analyses of bronchoalveolar lavage (BAL) cells from smokers with normal lung function (*n* = 25) and early stage COPD patients (*n* = 18). Multivariate modeling, pathway enrichment analysis, and correlation with clinical characteristics were performed to identify specific proteins and pathways of interest.

**Results:**

More pronounced alterations both at the protein- and pathway- levels were observed in female COPD patients, involving dysregulation of the FcγR-mediated phagocytosis-lysosomal axis and increase in oxidative stress. Alterations in pathways of the phagocytosis-lysosomal axis associated with a female-dominated COPD phenotype correlated well with specific clinical features: FcγR-mediated phagocytosis correlated with FEV_1_/FVC, the lysosomal pathway correlated with CT < −950 Hounsfield Units (HU), and regulation of actin cytoskeleton correlated with FEV_1_ and FEV1/FVC in female COPD patients. Alterations observed in the corresponding male cohort were minor.

**Conclusion:**

The identified molecular pathways suggest dysregulation of several phagocytosis-related pathways in BAL cells in female COPD patients, with correlation to both the level of obstruction (FEV_1_/FVC) and disease severity (FEV_1_) as well as emphysema (CT < −950 HU) in women.

**Trial registration:**

No.: NCT02627872, retrospectively registered on December 9, 2015.

**Electronic supplementary material:**

The online version of this article (10.1186/s12931-017-0699-2) contains supplementary material, which is available to authorized users.

## Background

Chronic obstructive pulmonary disease (COPD) is reported to be a leading cause of mortality worldwide and represents an important socioeconomic burden [1, 2]. Cigarette smoking is the most common etiology of COPD. Habitual cigarette smoking causes chronic inflammation in the small airways and lung parenchyma, leading to narrowing of the small airways and destruction of the alveolar walls [3]. A number of studies have demonstrated pronounced gender differences in susceptibility, respiratory symptoms, and lung function as well as in molecular markers of inflammation in COPD [4–6], with a higher frequency of hospitalization and mortality among women [7, 8]. Female smokers are more prone to lung function reduction [6] with reports of up to 50% higher risk of developing COPD compared to men after correction for smoking history [8–10]. However, the mechanisms underlying these gender-related differences are not well understood. Proteomics has deepened our understanding of the molecular pathogenesis of COPD in recent years using samples from different lung compartments [11, 12] [13, 14]. However, in spite of known gender differences in the clinical presentation, very few studies have investigated molecular gender differences. In our Karolinska COSMIC cohort, we have previously reported molecular gender differences in COPD in several compartments, including the BAL cell proteome using two-dimensional differential gel electrophoresis (2D–DIGE) [15]. Here we performed iTRAQ-label based shotgun proteomics to investigate immune cells from the lung (BAL cells). Shotgun proteomics facilitates investigation of a different proteome compartment than the previously reported 2D–DIGE approaches, and thus provides complementary information about the BAL cell proteome alterations due to smoking and COPD. Emphasis on the investigation reported here is on specific proteins and pathways related to pathological alterations in smoking-induced COPD. Results related to the effects of smoking prior to disease manifestations are reported in a companion paper [16].

## Methods

Detailed methods are provided in the Additional file 1.

### Study subjects and design

**Fig. 1:**
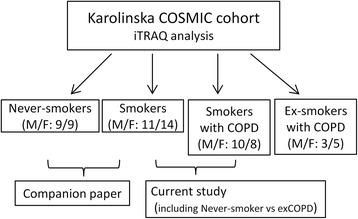
Flow chart outlining the study design and emphasis groups of the current vs. the companion paper [16]. A total of 69 subjects from the Karolinska COSMIC cohort, well-matched in terms of age, gender, and lung functions, were selected for iTRAQ proteomic investigations, including 18 healthy Never-smokers (9 male, 9 female), 25 Smokers with normal lung function (11 male, 14 female), 18 current smokers with COPD (10 male, 8 female), and 8 ex-smokers with COPD (3 male, 5 female). This manuscript focus on the alterations in proteomes and pathways related to COPD pathology (i.e., comparisons of Smoker vs. smokers with COPD, to some extent related to comparisons of healthy Never-smokers vs. ex-smokers with COPD (exCOPD)). The companion paper focuses on the effects of smoking prior to disease presentation, i.e., comparison of the Never-smoker vs Smoker groups [16]

This study was carried out on subjects from the Karolinska COSMIC cohort (ClinicalTrials.gov identifier NCT02627872) [15, 17–23], a three group cross-sectional study consisting of age- (45–65 years) and gender-matched groups of healthy never-smokers (Never-smokers), smokers with normal lung function (Smokers), and COPD patients (GOLD stage I-II, FEV1 > 50% and FEV_1_/FVC < 0.7), consisting of both current smokers (COPD) and ex-smokers (exCOPD, > 2 years since smoking cessation). Sixty-nine subjects were selected for iTRAQ proteomics analysis (Fig. 1), with results related to COPD pathology reported here, and results related to the effects of smoking reported in the companion paper. Smokers were matched in terms of smoking history (>10 pack years; > 10 cigarettes/day the past 6 months). All subjects underwent spirometry and high resolution CT [18, 21]. Participants had no history of allergy (negative IgE tests) or asthma, did not use inhaled or oral corticosteroids, and had no exacerbations for >3 months prior to study inclusion. The study was approved by the Stockholm regional ethical board (Case no. 2006/959–31/1) and informed written consent was obtained from all subjects. BAL cell samples were collected during bronchoscopy as previously described [24]. BAL T-cell subtypes were quantified using flow cytometry [17, 20].

### Proteomic analysis

Trypsinized protein extracts from 1.5 × 10^6^ BAL cells were labeled with 4-plex iTRAQ reagents, with the 114 isobaric tag dedicated to a pooled reference sample used for ratiometric normalization to reduce the variance between batches [25], while the subject samples were randomized and labeled with the 115, 116 or 117 isobaric tags. Labeled peptides were fractionated into 5 mix-mode fractions, and analyzed on an LTQ-Orbitrap Velos Pro (Thermo Scientific, Sunnyvale, California, USA) connected to a Dionex Ultimate NCR-3000RS (LC system, Sunnyvale, California, USA). Full scan MS spectra were acquired with resolution *R* = 120,000 at m/z 400. Peak integration of iTRAQ MS/MS spectra was performed by Proteome discoverer 2.1 (Thermo Fisher Scientific) searched against the UniProt human database (2015_12). Ratio data of samples to reference was log2 transformed.

### Statistical analyses

**Fig. 2:**
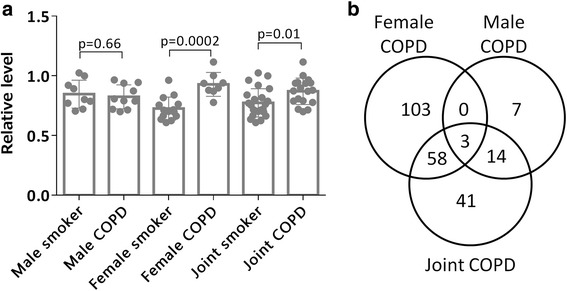
**a**) The protein (Isochorismatase domain-containing protein 2, ISOC2, Uniprot: Q96AB3) exemplifies the profile of a protein that was identified as significantly altered in the joint gender analysis (*p* = 0.01), in spite of a high heterogeneity (*I*^*2*^ = 0.88). Stratification by gender revealed that this significance was completely driven by that of in female population (*p* = 0.0002), and was not altered in males (*p* = 0.66) **b**) Venn diagram showing the overlap in alterations of proteomic profiles between Smokers and COPD groups in joint gender and gender stratified univariate statistical analyses. Only three proteins were altered in both male and female COPD patients, and the majority of protein alterations in female patients were unique

Univariate statistical analysis was performed using Student’s t-test (*p* < 0.05), followed by correction for multiple hypothesis testing according to Storey (q-value) [26]. The level of heterogeneity of the protein expression between genders for the comparison of Smoker vs COPD groups was quantified by means of *I*^*2*^ in order to determine if gender stratified statistical analysis was appropriate [27]. The heterogeneity tests indicated that 35 of 142 significantly altered proteins in the joint gender model displayed moderate-to-high heterogeneity between genders (I^2^ > 0.50, Additional file 2: Table S1), meaning that the majority of proteins in the joint gender model were driven by differences in one gender, as exemplified by ISOC2 (Fig. 2a).

Multivariate statistical modeling was performed using SIMCA 14.0 (Umetrics, Umeå, Sweden) including principal component analysis (PCA) and orthogonal projection to latent structure-discriminant analysis (OPLS-DA) [28]*.* In contrast to the more commonly used PCA modeling, OPLS analysis is a supervised method designed to separate structured noise unrelated (orthogonal, often intra-group variance) to the predictive variance of interest (e.g., COPD patients vs healthy subjects). The resulting “noise filter” increases the interpretability of the multivariate model, particularly in deriving the observed group separation back to the specific proteins driving the separation. For more information of how to interpret these models, or the related model statistics, please see [29]. Proteins with an absolute value of the scaled loadings of the first predictive component (|p(corr)[1]|) greater than the critical value of the Pearson correlation coefficient (*p* < 0.05) was considered significant for OPLS-DA models.

Model performance is reported as the goodness of fit (R^2^), the goodness of prediction based on 7-fold cross-validation (Q^2^), *p*-value for cross-validated ANOVA (CV-ANOVA) [29] and 200-fold permutation test [30, 31]. OPLS-DA models with R^2^ or Q^2^ greater than 0.9 and 0.5 respectively, were considered as good model, [30]. *P*-value of CV-ANOVA less than 0.05 were considered to be significant [31]. Multivariate correlation analysis of clinical- and demographical data with proteins was performed using partial least squares regression (PLS).

Pathway enrichment analysis was performed based on proteins found to be significantly altered in OPLS-DA models comparing female Smoker and COPD groups using KOBAS 2.0 [32], with pathway enrichment analysis performed based on the KEGG pathways database [33].

## Results

Clinical characteristics are summarized in Table 1. No significant differences were observed between Smoker and COPD groups in either gender (Table 1), except for the reductions of FEV_1_ (*p* < 0.01) and FEV_1_/FVC (*p* < 0.01) in COPD, as part of the cohort design.

**Table 1:** Clinical characteristics of Smokers and COPD groups, stratified by gender

	Male			Female		
	Smoker (*n* = 11)	COPD (*n* = 10)	*p*-value	Smoker (*n* = 14)	COPD (*n* = 8)	*p*-value
GOLD (I/II)	n.a.	4/6	n.a.	n.a.	5/3^b^	n.a.
GOLD (A/B)	n.a.	8/2	n.a.	n.a.	7/1^b^	n.a.
Age	55.2 ± 6.0	56.9 ± 6.3	NS	57.1 ± 5.5	58.4 ± 3.9	NS
BAL macrophages [%]	95.7 ± 2.4	97.0 ± 1.4	NS	96.4 ± 2.5	95.1 ± 2.8	NS
BAL lymphocytes [%]^a^	2.3 (1.2, 5.4)	1.4 (0.2, 5.6)	NS	1.7 (1, 7.2)	3.3 (1, 8.6)	NS
BAL neutrophils [%]^a^	0.8 (0.2, 3.6)	0.8 (0, 1.6)	NS	0.5 (0, 2.8)	0.5 (0.2, 1.4)	NS
BAL eosinophils [%]^a^	0.0 (0, 1.4)	0.3 (0, 1.2)	NS	0.1 (0, 1.2)	0.2 (0, 0.6)	NS
BAL basophils [%]^a^	0.0 (0, 0.4)	0.0 (0, 0)	NS	0.0 (0, 0.2)	0.0 (0, 0.2)	NS
BAL mast cells/10 vis fields^a^	3(0.8)	3(0.6)	NS	3(0.13)	1.5(0.20)	NS
BMI	25.4 ± 2.8	25.1 ± 3.9	NS	24.8 ± 2.6	24.7 ± 4.2	NS
Packs/years	41.4 ± 18.8	42.3 ± 10.1	NS	37.6 ± 11.4	37.3 ± 9.4	NS
Cigarettes/day in last 6 months	19.4 ± 7.8	18.4 ± 5.2	NS	15.6 ± 5.8	14.9 ± 7.9	NS
FEV_1_ (% predicted)	104 ± 12.1	76.0 ± 8.0	< 0.01	107 ± 14.12^c^	81.5 ± 14^b^	< 0.01
FEV_1_/FVC (% predicted)	77.3 ± 5.0	61.8 ± 6.6	< 0.01	77.4 ± 5.3^c^	58.8 ± 6.6^b^	< 0.01

Data are presented as mean ± SD tested by using t-test; or ^a^, median (minimum, maximum) for skewed data, tested by Mann-Whitney U test; n.a., not applicable; NS, not significant (*p* > 0.05); ^b^, no significant difference between male and female COPD patients (*p* > 0.30); ^c^, no significant difference between male and female smokers (*p* > 0.70)

### Proteome alteration in smokers with COPD

Univariate statistical analysis comparing smokers with normal lung function (Smokers) vs. current-smoker COPD patients (COPD) revealed 142 significantly altered proteins (*p* < 0.05,q < 0.30; Additional file 2: Table S1) Given the high heterogeneity scores (see Methods), statistical analyses were also performed following stratification by gender.

In females, 164 proteins were significantly altered in Smoker vs. COPD groups (*p* < 0.05, q < 0.30, Additional file 2: Table S6). In the corresponding males, 24 proteins (*p* < 0.05) were significantly altered, none of which passed correction for multiple testing (q < 0.30, Additional file 2: Table S6). Few altered proteins overlapped between the joint gender and gender stratified models, , and more than half of the significantly altered proteins were uniquely altered in female COPD patients (Fig. 2b).

**Fig. 3:**
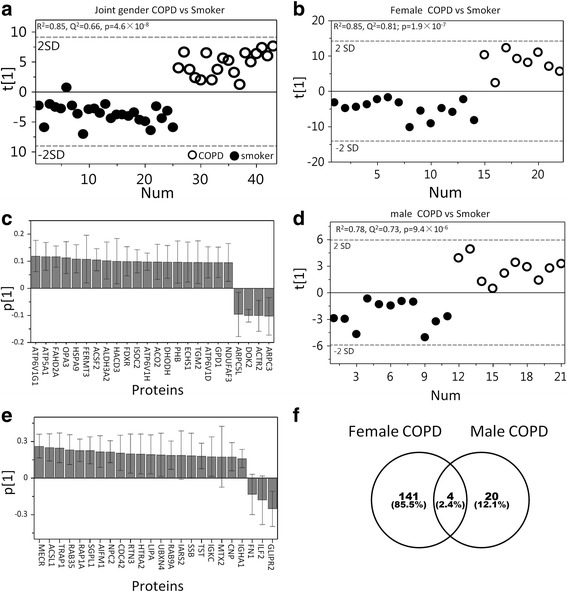
Multivariate OPLS-DA models comparing Smokers vs COPD groups before and after stratification by gender. OPLS-DA modeling showed significant separations between Smoker and COPD groups for **a**) joint gender (R^2^ = 0.85, Q^2^ = 0.66, p[CV-ANOVA] = 4.6 × 10^−8^, 116 proteins), **b**) females (R^2^ = 0.85, Q^2^ = 0.81; *p* = 1.9 × 10^−7^, 145 proteins) and **d**) males (R^2^ = 0.78, Q^2^ = 0.73, *p* = 9.4 × 10^−6^, 24 proteins). However, the predictive performances (Q^2^) were better following stratification by gender for both gender models. **c**) Loadings of the top 24 proteins out of 145 significant variables in the female COPD vs Smoker model; **e**) All 24 significant proteins from the male COPD vs Smokers model. There was no overlap among 24 top proteins of both gender models. A comprehensive list of loadings along with protein names and statistics are provided in Additional file 2: Table S2. **f**) Venn diagram displaying overlap between genders in protein alterations due to COPD based on the OPLS-DA models displayed in **b**) and **d**). Only four proteins (Q9NSE4, P02751, O95470, and P01876) were altered in both male and female smokers with COPD

Subsequent OPLS-DA multivariate modeling gave a good separation between joint gender Smoker and COPD groups (Fig. 3a; R^2^ = 0.85, Q^2^ = 0.66, p[CV-ANOVA] = 4.6 × 10^−8^) with 116 proteins driving the separation (|(p(corr)[1]| > 0.32, Additional file 2: Table S2). In concordance with the heterogeneity analysis (Additional file 2: Table S1), stratification by gender revealed a significant difference in the proteome alterations due to COPD between men and women, with very little overlap between genders. In the female population, 145 significantly altered proteins (|p(corr)[1]| > 0.45, Fig. 3c, Additional file 2: Table S2) drove the significant OPLS-DA model (Fig. 3b; R^2^ = 0.85, Q^2^ = 0.81; p[CV-ANOVA] = 1.9 × 10^−7^). In males, 24 proteins (Fig. 3e) drove the significant OPLS-DA model (Fig. 3d; R^2^ = 0.78, Q^2^ = 0.73, p[CV-ANOVA] = 9.4 × 10^−6^), with only 4 proteins overlapping between genders (Fig. 3f).

### Pathway enrichment analysis

**Fig. 4:**
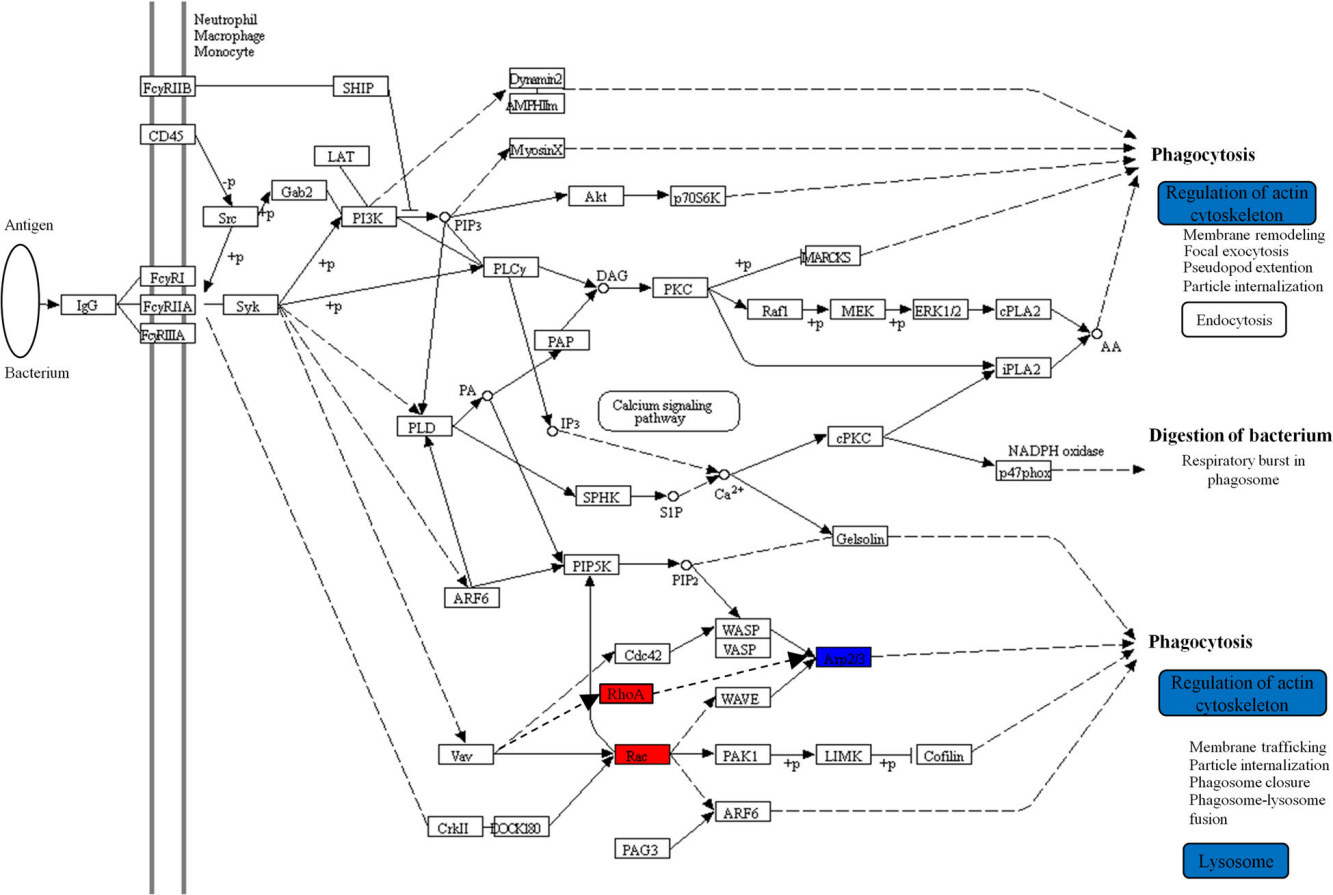
Dysregulation of FcγR-mediated phagocytosis in female COPD patients. The protein levels of ARPC4, ARPC5, ARPC5L, ARPC1B, ARPC2, ARPC3 decreased (blue) and Rac as well as RHOA increased (red) in BAL cells. Rearrangement of the actin cytoskeleton is a necessary driving force for FcγR-mediated phagocytosis [41, 42]. The decreased levels of Arp2/3 and actin cytoskeletal processes may thus imply that FcγR-mediated phagocytosis was hampered in spite of up-regulations of Rac and RhoA in COPD patients. The majority of proteins in the downstream regulation of actin cytoskeleton- and lysosome pathways were down-regulated in female COPD patients (Additional file 2: Table S3) Blue: down-regulated; red: up-regulated. This figure was created with KEGG pathway tool with minor modification

Pathway enrichment analysis for female Smoker vs COPD groups revealed 6 significantly enriched pathways (q < 0.05, Table 2, Additional file 2: Table S3): oxidative phosphorylation, citrate cycle, glutathione metabolism, FcγR-mediated phagocytosis, lysosomal pathway and regulation of actin cytoskeleton. No pathways were found to be significantly enriched in male Smoker vs. COPD groups. Accordingly, pathway enrichments found in the corresponding joint gender comparison was driven primarily by alterations in the female cohort (Table 2). The majority of proteins in the metabolic pathways, including oxidative phosphorylation, citrate cycle, amino acids metabolism, fatty acid metabolism (Additional file 2: Table S3) and glutathione metabolism were up-regulated, while those in the FcγR-mediated phagocytosis (Fig. 4), regulation of actin cytoskeleton and lysosomal pathways were down-regulated in female COPD patients (Additional file 2: Table S3).

**Table 2:** Pathways affected in COPD patients compared to smokers with normal lung function

	Female Smoker vs COPD			Male Smoker vs COPD			Joint Smoker vs COPD		
Pathways (background No.)	Hits	*p*-value	pFDR^b^	Hits	*p*-value	pFDR^b^	Hits	*p*-value	pFDR^b^
Oxidative phosphorylation^a^ (211)	12	6.0 × 10^−6^	3.3 × 10^−4^				3	0.06	0.21
Citrate (TCA) cycle (64)	6	1.1 × 10^−4^	2.9 × 10^−3^				3	1.7 × 10^−3^	0.02
Glutathione metabolism (89)	6	5.7 × 10^−4^	0.01				1	0.30	0.50
FcγR-mediated phagocytosis (165)	7	2.6 × 10^−3^	0.03				5	5.0 × 10^−4^	8.8 × 10^−3^
Lysosome^a^ (222)	8	5.6 × 10^−3^	0.04				5	1.3 × 10^−3^	0.02
Regulation of actin cytoskeleton (387)	11	4.1 × 10^−3^	0.04				8	1.1 × 10^−4^	4.5 × 10^−3^
Phagosome (287)	8	1.5 × 10^−2^	0.10				5	4.3 × 10^−3^	0.04
Fatty acid metabolism (94)	4	2.1 × 10^−2^	0.13	2	7.8 × 10^−3^	0.29	3	5.4 × 10^−3^	0.05
Focal adhesion (310)				3	2.7 × 10^−2^	0.29	5	0.01	0.09
Proteasome							4	3.1 × 10^−4^	8.2 × 10^−3^
Endocytosis							7	1.9 × 10^−3^	0.03

Pathway enrichment analysis for female, male and joint gender comparisons of Smoker vs COPD groups were based on 145, 24 and 116 proteins driving the respective OPLS-DA models,. ^a^This pathway was detected in female COPD patients by two-dimensional difference gel electrophoresis (2D–DIGE) analysis [15]; More significant pathways were found in female COPD patients for iTRAQ-based proteomics is a platform with higher resolution and more sensitiveness than 2D–DIGE. ^b^FDR corrected p-value by Benjamini and Hochberg’s method; COPDs, smokers with COPD

Direct comparisons of female vs. male COPD patients was performed using OPLS-DA analysis, yielding 119 significant proteins (R^2^ = 0.92, Q^2^ = 0.86, p[CV-ANOVA] = 4.6 × 10^−7^) representing 7 significant pathways (*p* < 0.05), including oxidative phosphorylation, FcγR-mediated phagocytosis, lysosome and citrate cycle (Additional file 2: Table S4).

### Pathways correlate with lung function and emphysema

**Fig. 5:**
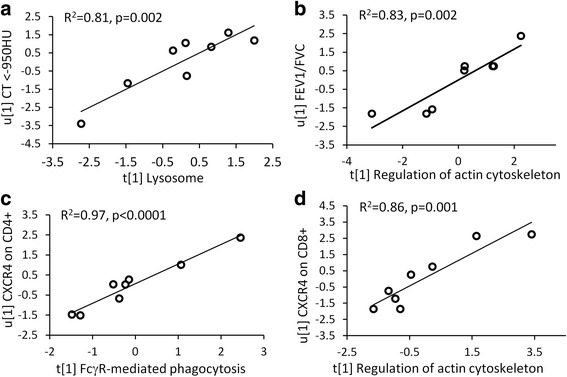
Correlations of the pathways of interest with clinical parameters and T-cell subpopulations in BAL in female COPD patients. **a**) Correlation between the proteins in the lysosomal pathway and CT attenuation values <−950 HU in female COPD group (PLS inner relation, R^2^ = 0.81, *p* = 0.002). **b**) The correlation between the proteins in the regulation of actin cytoskeleton pathway and FEV_1_/FVC (PLS inner relation, R^2^ = 0.83, *p* = 0.002) in female COPD patients. **c**) The proteins in the FcγR-mediated phagocytosis pathway highly correlated with chemokine receptor CXCR4 on CD4^+^ T cells (R^2^ = 0.97, *p* < 0.0001). **d**) The proteins in the regulation of actin cytoskeleton pathway correlated with CXCR4-expressing on CD8^+^ T cells (R^2^ = 0.86, *p* = 0.001)

CT data was acquired as previously described [18, 21]. The percentage of attenuation values <−950 HU was increased in female COPD patients compared to the female Smoker group (*p* = 0.007, Additional file 1: Figure S3), but not in the corresponding males. CT < −950 HU values significantly correlated with the lysosomal- (R^2^ = 0.81, *p* = 0.002; Fig. 5a) and glutathione metabolism (R^2^ = 0.66, *p* = 0.01) pathways in female COPD patients, but not in males. The proteins from the FcγR-mediated phagocytosis and regulation of actin cytoskeleton pathways correlated with FEV_1_/FVC (R^2^ = 0.54, *p* = 0.04; R^2^ = 0.83, *p* = 0.002, Fig. 5b; respectively), while the proteins from the regulation of actin cytoskeleton and oxidative phosphorylation pathways correlated with FEV_1_ in female COPD patients (R^2^ = 0.51, *p* = 0.05; R^2^ = 0.52, *p* = 0.04; respectively). No significant correlations were found in the female Smoker or male groups.

### Pathways correlated with T-cell subsets

The proportion of CD4^+^ and CD8^+^ T-cell, as well as their subtypes expressing chemokine receptors CXCR3, CXCR4 and CCR5, or the activation marker CD69 in the BAL cell populations were quantified in the BAL cell population using flow cytometry [17, 20]. Protein abundances in the FcγR-mediated phagocytosis, regulation of actin cytoskeleton, and lysosomal pathways correlated with the proportion of CD4^+^CXCR4^+^ T-cells (R^2^ = 0.97, *p* < 0.0001, Fig. 5c; R^2^ = 0.93, *p* < 0.0001; R^2^ = 0.61, respectively) as well as with CD8^+^CXCR4^+^ T-cells (R^2^ = 0.77, *p* = 0.009; R^2^ = 0.86, *p* = 0.001, Fig. 5d; R^2^ = 0.86, *p* = 0.001, respectively). Proteins from the lysosomal pathway also correlated significantly with the overall proportion of CD8^+^ and CD4^+^ T cells (R^2^ = 0.58, *p* = 0.03; R^2^ = 0.57, p = 0.03, respectively), as well as CXCR3 on CD8^+^ (R^2^ = 0.66, *p* = 0.01) and CD4^+^ (R^2^ = 0.68, p = 0.01) cells. The corresponding correlations in female Smokers and male groups were weaker or not significant.

### iTRAQ proteomics versus 2D–DIGE proteomics

**Fig. 6:**
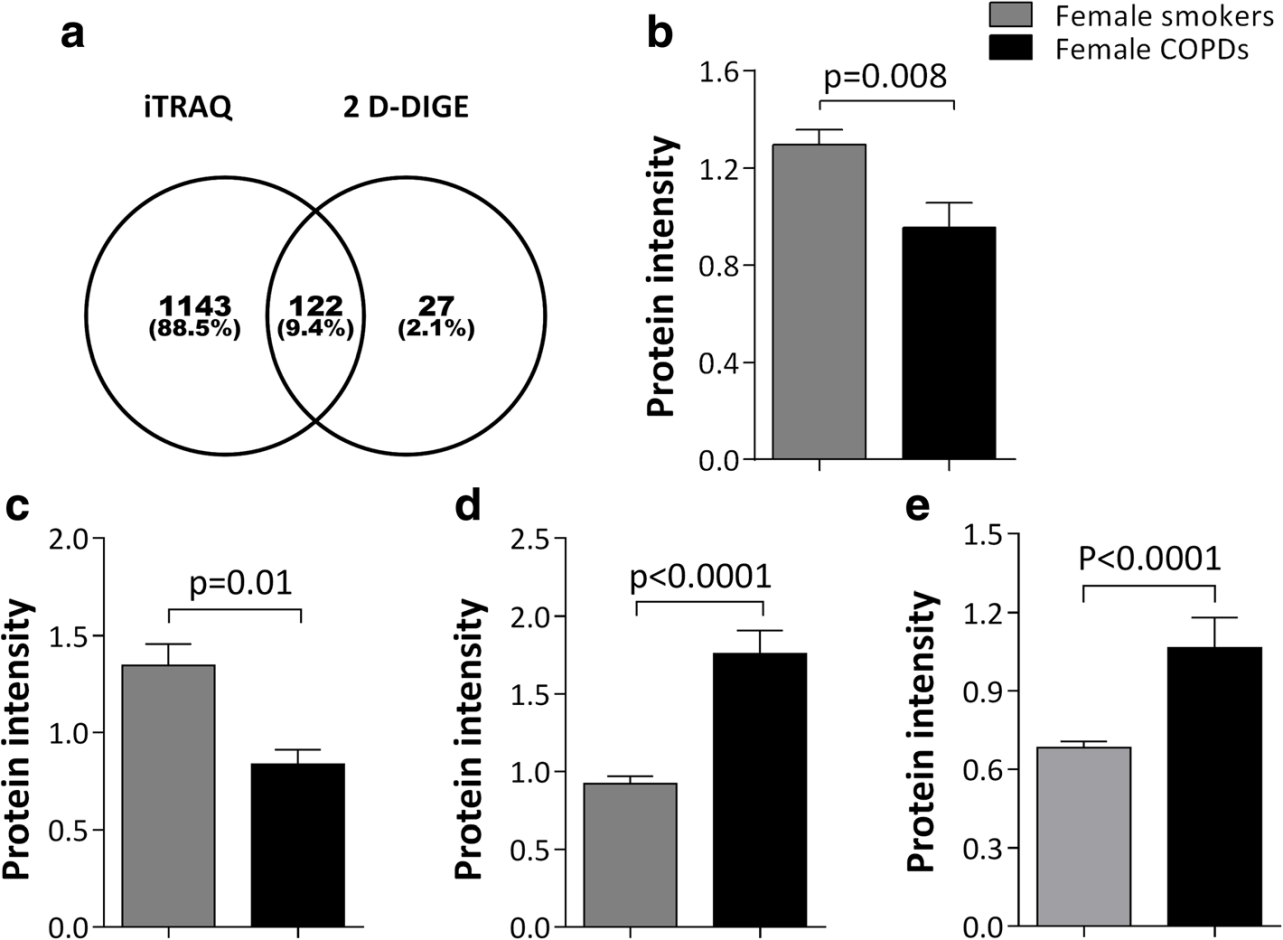
Comparison of results from 2D–DIGE intact proteomics [15] and iTRAQ shotgun proteomics analysis on the same cohort. **a**) Venn diagram showing that the majority of protein species identified by 2D–DIGE overlapped with proteins identified by iTRAQ. Ninety percent of the proteins identified by iTRAQ were not identified by 2D–DIGE. However, several of the proteins identified by 2D–DIGE served as validation of key proteins in pathways found to be enriched in the current study: Actin-related protein 3 (ARP3; panel **b**;, 2D–DIGE: *p* = 0.008; iTRAQ: *p* = 0.01, is involved in the pathways FcγR-mediated phagocytosis and regulation of actin cytoskeleton; Beta-hexosaminidase subunit beta (HEXB; panel **c**;)2D–DIGE: *p* = 0.01; iTRAQ: *p* = 0.02,); and ATP synthase subunit beta (ATP5B, panel **d**;) (2D–DIGE: *p* < 0.0001; *p* = 0.04,) are both involved in the lysosomal- and oxidative phosphorylation pathways,; **e**), Leukotriene A-4 hydrolase (LTA4H) (2D–DIGE: p < 0.0001; iTRAQ: *p* = 0.01,). is one of the most prominent proteins for driving the separation between female COPD and Smokers groups by both proteomics platforms. Data in b, c, d and e are expressed as mean ± SE

2D–DIGE analyses have been performed on the BAL samples from the same cohort [15]. Ninety percent of the proteins identified by iTRAQ were novel, i.e. not previously identified by 2D–DIGE (Fig. 6a). Among the few overlapping proteins, several key proteins present in the pathways identified as significantly enriched through the iTRAQ analyses were found, and the 2D–DIGE data set thereby serves as a technical validation for the iTRAQ analyses. Some of these key proteins are presented in Fig. 6: the levels of protein ARP3 (Fig. 6b) involved in the pathways FcγR-mediated phagocytosis and regulation of actin cytoskeleton, and HEXB (Fig. 6c) involved in the lysosomal- and oxidative phosphorylation pathways, decreased in both proteomics platforms, whereas ATP5B (Fig. 6d) also involved in the lysosomal- and oxidative phosphorylation pathways, increased in both platforms. lLTA4H (Fig. 6e) increased in female COPD patients in both studies. The full list of proteins identified by iTRAQ is presented in Additional file 2: Table S5.

## Discussion

COPD is a heterogeneous inflammatory disease manifesting itself in a multitude of sub-phenotypes that are likely to involve distinct molecular pathways in the disease development. Smoking-related COPD is the most common sub-phenotype. Smoking women experienced a worse decline in lung function and a higher risk of hospitalization than smoking men even after adjustment for smoking history in large cohort studies [9, 10], with the accelerated decline in lung function being particularly pronounced after menopause [34]. By means of iTRAQ-based analyses, clear differences in the BAL cell proteomes of smokers with COPD compared with Smoker with normal lung function of both genders were demonstrated, with more pronounced alterations in female COPD patients. The low level of overlap between genders indicates distinct molecular gender difference in COPD, thereby supporting our previous findings at the proteome [15], lipidome [19], and metabolome levels [22]. Consistently, these differences were apparent also at the pathway level, with more pronounced alterations in female COPD patients, involving phagocytosis-related process (FcγR-mediated phagocytosis, regulation of actin cytoskeleton, and lysosome), and oxidative stress (oxidative phosphorylation, and glutathione metabolism). In contrast, the gender differences observed between smokers with normal lung function and healthy never-smokers were minor (see Companion manuscript [16]). As such, the gender differences observed here appear to be isolated to COPD pathology.

Phagocytosis represents an important first line of defense for clearance of invading organisms and protection from infections in the lung, as well as clearance of particulate matter and debris resulting from cigarette smoking [35]. The ligand of Fcγ-receptors, IgG, coats the surface of pathogens such as viruses, bacteria, and fungi, which facilitates recognition by and binding to Fcγ-receptors, thereby initiating phagocytosis. Defective phagocytosis of bacteria by alveolar macrophages in COPD patients has been associated with bacterial colonization in the airway [36, 37] and increased risk of exacerbations of COPD, which is further associated with an accelerated progression of airflow obstruction [38]. Non-opsonic mediated phagocytosis has been proposed to be predominated in COPD [35, 37]. However, our results suggested dysfunction also of the opsonic pathways, with dysregulation of FcγR-mediated phagocytosis primarily in female COPD patients. This may afford an explanation as to why female smoker with COPD have a higher risk of hospitalization and exacerbations [39, 40], both frequently linked to infections [9]. FcγR-mediated phagocytosis was altered also in ex-smoker COPD patients (Additional file 1: Figure S1), indicating that the dysregulation may persist even following smoking cessation in female COPD patients.

Rearrangement of the actin cytoskeleton is necessary for phagocytosis and engulfment of foreign particles [41, 42]. The phagosome fuses with the lysosome to form a phagolysosome for digestion. Here we observed dysregulation not only in the lysosomal pathway as previously described [15], but also in two upstream pathways of phagocytosis and regulation of actin cytoskeleton in female COPD patients. Proteins in FcγR-mediated phagocytosis and regulation of actin cytoskeleton correlated with the level of obstruction, suggesting that these two pathways play a more prominent role in the pathogenesis of COPD in females. Proteins of the lysosomal pathway also significantly correlated with CT attenuation values <−950 HU, an index of the proportion of emphysema [43], in female but not male COPD patients, indicating that dysregulation of the lysosomal pathway is associated with alveolar destruction primarily in female COPD patients.

The three pathways related to the phagocytosis-lysosomal axis also highly correlated with CXCR4^+^ CD4+ and CD8+ T-cells in BAL in female COPD patients (Fig. 5). CXCR4 specifically binds to CXCL12 [44], and CXCL12/CXCR4 interaction controls the homeostatic localization, development, and polarization of immune cells in peripheral tissues, as well as their migration under inflammatory conditions [44]. Furthermore, the chemotaxis of CXCL12/CXCR4 interaction is regulated by Rho GTPases including RhoA, Rac1, both of which were upregulated in FcγR-mediated phagocytosis and regulation of actin cytoskeleton pathways in female COPD patients. Activation of Rho GTPases inhibit T-cell polarization and migration [45] mediated by CXCL12/CXCR4, indicating defective phagocytosis along with dysregulation of T-cell polarization and migration in female COPD patients.

The downstream lysosomal pathway was altered in smokers with normal lung function as compared to healthy never-smokers of both genders [16]. The selective alterations of the lysosomal pathway along with two upstream pathways in females but not male COPD patients further suggests their role in the molecular gender differences in COPD pathogenesis.

Oxidative phosphorylation is the source of reactive oxygen species (ROS), and glutathione is a key antioxidant against the damage of oxidative stress induced by ROS. The increases in oxidative phosphorylation and glutathione metabolism also stress the importance of these two pathways in disease pathology in COPD. Detailed discussion in the Additional file 1.

Even though the Karolinska COSMIC cohort represents a large study within the scope of sampling by bronchoscopy, the group sizes following stratification by gender and current smoking status [46] are relatively small, making it difficult to generalize the findings. However, the molecular alterations in female COPD patients observed at several molecular levels from multiple lung compartments in this cohort, including metabolomes [22] and oxylipins [19] in airway exudates and serum, as well as proteomes in lung immune- [15] and epithelial [47] cells, with significant overlap in the molecular pathways identified provide added validity to these findings.

## Conclusion

In conclusion, analyses of the BAL immune cell proteome using iTRAQ proteomics revealed gender-specific proteome alterations due to COPD, with very limited overlap between male and female COPD patients. In women, the altered pathways involved dysfunction in FcγR-mediated phagocytosis, regulation of actin cytoskeleton, lysosomal, and oxidative stress pathways that correlated with the degree of obstruction, and emphysema. No alterations were observed in the male cohort. Furthermore, the gender-specific dysregulation of the phagocytosis-lysosomal axis and T-cell polarization may provide mechanistic clues to the faster decline of lung function and higher risk of hospitalization observed in female COPD patients. Given the lack of gender differences in smokers with normal lung function (see companion paper RERE-D-17-00418.1, with DOI:10.1186/s12931-017-0695-6 12931_2017_695), our results suggest that the pathogenesis of COPD differs in female and male smokers in early disease stages. These results also stress the importance of gender stratification both in terms of elucidation the mechanisms underlying smoking-induced COPD, as well as for the development of relevant diagnosis and treatment strategies for COPD.

## Additional files

Additional file 1: Supplementary Methods. **Figure S1.** Analysis of Share and Unique Structure (SUS) between OPLS-DA models of female Smoker vs COPD (x-axis) and Never-smoker vs ex-smoker with COPD (exCOPD) (y-axis). **Figure S2**. Multivariate sensitivity analysis of the impact of menopausal status on proteomic profiling in female COPD patients. **Figure S3.** The percentage of CT attenuation values <−950 HU in the Smoker and COPD groups, stratified by gender. (DOC 1849 kb)

Additional file 2: **Table S1.** Heterogeneity indeces (I^2^) for proteins significantly altered between Smoker vs COPD groups joint gender as well as gender stratified models. **Table S2.** Protein identities and model statistics of proteins of interest from OPLS-DA models comparing Smoker and COPD groups for joint gender as well as gender stratified models. **Table S3.** Uniprot accessions, gene names, protein names, as well as and direction of alteration for proteins involved in pathways significantly altered due to COPD. **Table S4.** Pathways significantly enriched in female vs male COPD patients. **Table S5.** Names and MS/MS data of proteins used in statistical analyses. **Table S6.** Significantly altered proteins i Smoker vs COPD groups, stratified by gender. (XLSX 338 kb)

## Abbreviations

2D–DIGE: Two-dimensional difference gel electrophoresis
BAL: Bronchoalveolar lavage
COPD: Chronic obstructive pulmonary disease
FDR: False discovery rate
HU: Hounsfield Units
iTRAQ: Isobaric tags for relative and absolute quantitation
KEGG: Kyoto encyclopedia of genes and genomes
OPLS-DA: Orthogonal projection to latent structure-discriminant analysis
p(corr)[1]: Scaled loadings of the predictive component of OPLS-DA model
p[CV-ANOVA]: *p*-value of cross-validated ANOVA
PCA: Principal component analysis
pFDR: Corrected p-value using Benjamini and Hochberg’s FDR method
PLS: Partial least squares correlation analysis
Q^2^: Goodness of prediction (based on 7-fold cross-validation)
R^2^: Goodness of fit (determination coefficient)
SUS plot: the plot of shared and unique structure
