# Supplementary material for: Proteomic profiling of lung immune cells reveals dysregulation of phagocytotic pathways in female-dominated molecular COPD phenotype
Source: Respir Res. 2018 Mar 8;19:39. doi: 10.1186/s12931-017-0699-2 (PMC5842633; doi:10.1186/s12931-017-0699-2)
Supplement: Supplementary file 1 — Supplementary Methods. Figure S1. Analysis of Share and Unique Structure (SUS) between OPLS-DA models of female Smoker vs COPD (x-axis) and Never-smoker vs ex-smoker with COPD (exCOPD) (y-axis). Figure S2. Multivariate sensitivity analysis of the impact of menopausal status on proteomic profiling in female COPD patients. Figure S3. The percentage of CT attenuation values <−950 HU in the Smoker and COPD groups, stratified by gender. (DOC 1849 kb) [file 12931_2017_699_MOESM1_ESM.doc]

# Methods

**Study Subjects and design**

This study was carried out on subjects from the Karolinska COSMIC cohort (ClinicalTrials.gov identifier NCT02627872) [^1-6^](#_ENREF_1). The Karolinska COSMIC study is a three group cross-sectional study in which each group was stratified by gender. The overall aim of the Karolinska COSMIC study is to investigate the differentiation between genders in response to smoking as well as early stage COPD by integrating several aspects of COPD and smoking through the use of imaging, transcriptomics, proteomics, metabolomics, and lymphocyte profiling in the context of clinical phenotypes. The study includes three age- (45-65 years) and gender matched groups of healthy never-smokers (Never-smokers), smokers with normal lung function (Smokers), and smokers with mild to moderate disease COPD (GOLD stage I-II, FEV1>50% and FEV_1_/FVC<0.7), consisting of both current smokers (COPD) and ex-smokers (exCOPD, >2 years since smoke cessation). A total of 69 subjects, evenly distributed across the groups (Figure 1), were selected for iTRAQ proteomics analysis as described below. The results reported here focus on alterations related to COPD pathology, with comparison of the Smokers (n=25, male/female: 11/14) and current-smoker COPD patients (n=18, male/female: 10/8) at the forefront (Table 1), with some comparisons of the Never-smoker (n=, male/female: 8/9) and ex-smoker COPD (n=8 male/female: 3/5). Results related to the effects of smoking are reported in the companion paper.

Smoking COPD patients and smokers were matched in terms of smoking history (>10 pack years and >10 cigarettes/day the past 6 months). All subjects were well characterized clinically including spirometry and high-resolution computed tomography (CT) . Participants had no history of allergy or asthma did not use inhaled or oral corticosteroids and had no exacerbations for at least 3 months prior to study inclusion. In vitro screenings for the presence of specific IgE antibodies (Phadiatop; Pharmacia Corp) were negative. Reversibility was tested after inhalation of two doses of 0.25 mg terbutaline (Bricanyl; Turbuhaler**®**; AstraZeneca). Medications (including oral contraceptives, estrogen replacement and NSAIDs or other potential lipid mediator-modifying drugs) were recorded by means of a questionnaire. Lung function parameters were calculated as post-bronchodilator percent of predicted using the European Community of Coal and Steel (ECCS) normal values. The study was approved by the Stockholm regional ethical board (Case no. 2006/959-31/1) and informed written consent was obtained from all subjects. The experiments conformed to the principles in the WMA Declaration of Helsinki. BAL cell samples were collected during bronchoscopy as previously described [^7^](#_ENREF_7). BAL T-cell subtypes were quantified using flow cytometry as previously described .

**Protein sample preparation**

Approximately 1.5 × 10^6^ cells were pelleted and washed twice with PBS (w/Protease Inhibitor Cocktail (Roche, Mannheim, Germany)) before lysis with 125 µl ice-cold RIPA buffer (1% NP-40, 0,1% SDS, 0.5% Na DOC, 50 mM Tris, 150 mM NaCl, PH 7.4) containing protease inhibitor. The lysis was performed by 15 seconds vortexing, 15 seconds sonication followed by 10 minutes incubation on ice, repeated 3 times. To remove cell debris the lysate was centrifuged at 8,000g for 10 minutes at 4ºC. The supernatant was stored in aliquots at -80ºC. Protein concentrations were determined using BCA protein assay kit (Thermo Fisher Scientific, Rockford, USA) according to the manufacture’s description. To remove interfering detergents 20 µg of each the protein samples was cleaned using the 2-D Clean-Up Kit (GE Healthcare, Uppsala, Sweden) according to the manufacture’s protocol and then stored at -80ºC prior to iTRAQ labeling.

Reference samples were made by pooling 10 µg from all 69 samples and divided into 20 µg aliquots and used as reference samples in all 23 iTRAQ experiments.

**Protein digestion and iTRAQ labeling**

All 69 protein samples and 23 references were reduced, cysteine blocked, trypsin digested (1:20, trypsin: protein, w/w), iTRAQ labeled (4-plex) and combined according to the protocol using the chemicals provided (AB Sciex), except for the following modifications: The content of one iTRAQ reagents was divided into two samples and the labeling reaction was increased to 1.5 hour. The reference sample in all experiments was labeled with the 114 isobaric tag, while the individual subject samples were randomized and labeled with the 115, 116 or 117 isobaric tags. The use of a pooled internal standard to reduce the technical variance between batches is a widely applied approach in proteomics.By dedicating one iTRAQ channel to a reference sample created by pooling from subjects from all study groups of interest (here: never-smokers, smokers, COPD patients of both genders) assures that i) that all proteins of interst for biomarker discovery or mechanistic invetigations are represented in the reference pool ii) the technical variance between batches can be corrected through by ratios normalization between sample and pooled reference.[^8^](#_ENREF_8)

**Mixed-mode fractionation**

iTRAQ labeled peptides were fractionated into 5 fractions using a mix-mode chromatography utilizing a Sielc Promix column (MP-10.250.0530, 1.0 × 250 mm, 5 μm, 300A, Sielc Technologies, Prospect Heights, Illinois, USA)), using an Agilent 1260 series LC system (Agilent Technologies, Palo Alto, CA). The peptides were reconstituted in 20 mM ammonium formate, 3% ACN (buffer A) and loaded on the column in 85% buffer A for 10 minutes at a flow rate of 50 µl/min. The peptides were eluted from the column increasing the contents of buffer B (2 mM ammonium formate, 80% ACN, pH 3.0), from 15 % to 60 % in 35 minutes and further to 100% buffer B over 10 minutes. Buffer B was held constant for 5 minutes before the column was equilibrated for 10 minutes in 85% buffer A. The fractions from the first 10 and the last 10 minutes of the gradient were discarded.

**LC-MS/MS analyses and MS/MS data preprocessing**

Five mix mode fractions from each iTRAQ experiment were analyzed on an LTQ-Orbitrap Velos Pro (Thermo Scientific, Sunnyvale, California, USA) equipped with a nanospray Flexion source (Thermo Fischer Scientific) and connected to a Dionex Ultimate NCR-3000RS (LC system, Sunnyvale, California, USA). The peptides were trapped on a pre-column (Dionex, Acclaim PepMap 100, 2 cm x 75 µm i.d, 3 µm C18 beads) in buffer A (2% ACN, 0,1% FA) at a flow rate of 5 µl/min for 5 minutes before separation by reverse phase chromatography (Dionex, Acclaim PepMap 100, 15 cm x 75 µm i.d., 3 µm C18 beads) at a flow of 280 nL/min. The mixed mode fractions were analyzed using two slightly different nano LC gradients. The first two fractions were run on a LC gradient starting at 5% buffer B (90% ACN, 0.1% FA) (0-6.0 min) ramping to 6.5% buffer B (6.0-6.5min), then to 20%B (6.5-50min), further to 35% B (50-67 min) and finally to 90% B (67-70 min). The three last fractions were analyzed on a slightly steeper gradient, going to 25% B (6.5-50 min) and to 38% B (50-67 min). Full scan MS spectra were acquired in the Orbitrap with resolution R = 120,000 at m/z 400. The 7 most intense eluting peptides above 1000 counts and charge state 2 or higher, were sequentially isolated in the linear ion trap. Fragmentation in the Higher-Energy Collision Dissociation (HCD) cell was performed with a normalized collision energy of 40% and activation time of 0.1 ms. Fragments were detected in the Orbitrap at a resolution of 15000.

Peak integration was performed using the method of most confident centroid with integration tolerance of 0.01Da. The iTRAQ MS/MS data was searched against the UniProt human database (2015_12) using Proteome discoverer 2.1 (Thermo Fisher Scientific) as a search engine. The search parameters were precursor mass tolerance 10 ppm, fragment mass tolerance 0.05 Da and a maximum number of missed cleavages was 2. Fixed modifications used were iTRAQ (K and N-terminus) and methyl thiosulfate (C). Prior to analysis, the ratio data of samples to reference was log2 transformed to avoid weighting based on protein abundance in the analyses.

**Statistical analyses**

Univariate statistical analyses were performed using Student’s t-test (p<0.05) and followed by the correction for multiple hypothesis testing according to Storey (q value) [^9^](#_ENREF_9). Quantification of heterogeneity of protein significance between both gender Smoker vs COPD groups was estimated by *I^2^* computed from Cochran’s Q test. *I^2^* values of 25%, 50%, and 75% were considered as the criteria of low, moderate and high heterogeneity [^10^](#_ENREF_10). Multivariate statistical modeling was performed using SIMCA 14.0 (Umetrics, Umeå, Sweden) including principal component analysis (PCA) and supervised orthogonal projection to latent structure-discriminant analysis (OPLS-DA) [^11^](#_ENREF_11). Protein with an absolute value of the scaled loadings of the first predictive component (|p(corr)[1]|) greater than the critical value of the Pearson correlation coefficient was considered significant for OPLS-DA models.

Model performance is reported as the goodness of fit (R^2^), the goodness of prediction based on 7-fold cross-validation (Q^2^), p-value for cross-validated ANOVA (CV-ANOVA) and Y-intercepts of regression lines for R^2^ and Q^2^ after 200 times of permutation test as previously suggested . Shared-and-unique structures (SUS) analysis [^14^](#_ENREF_14) was performed to investigate the effect of menopause.

## Pathway analysis

Subsets of proteins selected by multivariate OPLS-DA modeling were used to perform functional annotation and pathway analysis using KOBAS 2.0[^15^](#_ENREF_15) utilizing KEGG pathways.[^16^](#_ENREF_16) Multivariate correlation analysis of clinical- and demographical data and protein levels was performed at the pathway level using PLS. Correlations meeting the criteria of PLS inner relations with p<0.05 and a cumulative R^2^>0.5 (explained variance>50% ) were considered significant.

# Results

**Proteome alterations in COPD patients and gender difference**

In females, 3 subjects in the Smokers group were pre-menopausal, while all subjects in the COPD group were post-menopausal. To evaluate the effect of menopause, a new model excluding pre-menopausal individuals was fitted to perform multivariate sensitivity analysis against the original model. The correlation coefficient between the profiles with and without pre-menopausal subjects was R^2^=0.98 (e-Figure 2).

# Discussion

# About the role of oxidative phosphorylation, TCA and glutathione metabolism in pathogenisis of COPD

TCA cycle and oxidative phosphorylation are two key metabolic pathways with close linkage. The activities of oxidative phosphorylation and TCA cycle were increased in female Smokers with normal lung function compared to healthy never-smokers (Yang M. et al. companion manuscript) and further increased in female COPD patients compared to Smokers. Activities of these two pathways, influenced by both current cigarette consumption and COPD pathology, correlated with FEV_1_. Higher protein expression in oxidative phosphorylation was reported both in our own studies [^2^](#_ENREF_2) from the same cohort, as well as from others in bronchial epithelial cells in smoking COPD patients [^17^](#_ENREF_17). The elevated energy metabolism may represent a compensatory mechanism and may be early indicators of weight loss often observed in later stages of COPD. Increased activity of oxidative phosphorylation may also result in the accumulation of excessive levels of reactive oxygen species (ROS) and concurrent lung tissue damage . Glutathione is a key antioxidant present at millimolar levels in the lung and represents the first line of defense against the damage of oxidative stress induced by ROS, both intra- and extracellularly [^20^](#_ENREF_20). The increases in oxidative phosphorylation and two important antioxidant enzymes in glutathione metabolism, glutathione S-transferases κ1 and glutathione peroxidase 1, indicated that oxidative stress induced by smoking is aggravated in COPD in spite of a similar level of volitional exposure to first-hand cigarette smoke. A significant correlation between glutathione metabolism and FEV_1_/FVC further indicates the importance of these pathways in disease pathology in COPD.

Female gender has been proposed as a risk factor for susceptibility to the oxidative damage caused by cigarette smoking [^21^](#_ENREF_21). Female smokers in their 40’s and 50’s have a 50% increased risk of developing COPD compared to their male peers, even after correction for pack-years of smoking [^22^](#_ENREF_22), and women with airway obstruction are prone to an accelerated decline in lung function [^23^](#_ENREF_23). It has been suggested that down-regulation of antioxidant genes in smoking induced COPD in females may be the culprit: Tam and colleagues recently showed that long-term exposure to smoking was associated with down regulation of a range of antioxidant genes and increased oxidative stress in female, but not male or ovariectomized mice [^24^](#_ENREF_24). These effects, as well as associated increases in small airway remodeling and distal airway resistance, were attenuated by Tamoxifen treatment, indicating that female sex hormones play an important role in the sensitivity to smoking, with an impaired antioxidant defense being a contributing factor. The observed up-regulation of glutathione metabolism may be reflective of the launch of compensatory or protective mechanisms within macrophages in response to the overall increase in oxidative stress in female COPD patients. Increased oxidative stress primarily in female COPD patients was observed also at the systemic level in the Karolinska COSMIC cohort, with elevated levels of the oxidative stress markers hypoxanthine detected in the serum [^25^](#_ENREF_25). However, the strong correlation between the models constructed with and without pre-menopausal women (e-Figure 2) indicated that the altered proteomic profiles observed in these studies were not directly related to menopausal status, and other gender-related factors may also be involved.

# References

1. Forsslund H, Mikko M, Karimi R, et al. Distribution of T-cell subsets in BAL fluid of patients with mild to moderate COPD depends on current smoking status and not airway obstruction. *Chest.* 2014;145(4):711-722.

2. Kohler M, Sandberg A, Kjellqvist S, et al. Gender differences in the bronchoalveolar lavage cell proteome of patients with chronic obstructive pulmonary disease. *The Journal of allergy and clinical immunology.* 2013;131(3):743-751.

3. Karimi R, Tornling G, Forsslund H, et al. Lung density on high resolution computer tomography (HRCT) reflects degree of inflammation in smokers. *Respiratory research.* 2014;15:23.

4. Balgoma D, Yang M, Sjodin M, et al. Linoleic acid-derived lipid mediators increase in a female-dominated subphenotype of COPD. *The European respiratory journal.* 2016;47(6):1645-1656.

5. Forsslund H, Yang M, Mikko M, et al. Gender differences in the T-cell profiles of the airways in COPD patients associated with clinical phenotypes. *International journal of chronic obstructive pulmonary disease.* 2017;12:35-48.

6. Karimi R, Tornling G, Forsslund H, et al. Differences in regional air trapping in current smokers with normal spirometry. *The European respiratory journal.* 2017;49(1):Accepted.

7. Lofdahl JM, Cederlund K, Nathell L, Eklund A, Skold CM. Bronchoalveolar lavage in COPD: fluid recovery correlates with the degree of emphysema. *The European respiratory journal.* 2005;25(2):275-281.

8. Song X, Bandow J, Sherman J, et al. iTRAQ experimental design for plasma biomarker discovery. *Journal of proteome research.* 2008;7(7):2952-2958.

9. Storey JD. A direct approach to false discovery rates. *Journal of the Royal Statistical Society: Series B (Statistical Methodology).* 2002;64(3):479-498.

10. Higgins JP, Thompson SG, Deeks JJ, Altman DG. Measuring inconsistency in meta-analyses. *Bmj.* 2003;327(7414):557-560.

11. Bylesjö M, Rantalainen M, Cloarec O, Nicholson JK, Holmes E, Trygg J. OPLS discriminant analysis: combining the strengths of PLS-DA and SIMCA classification. *Journal of Chemometrics.* 2006;20(8-10):341-351.

12. Silva E, Souchelnytskyi S, Kasuga K, Eklund A, Grunewald J, Wheelock AM. Quantitative intact proteomics investigations of alveolar macrophages in sarcoidosis. *The European respiratory journal.* 2013;41(6):1331-1339.

13. Eriksson L, Trygg J, Wold S. CV-ANOVA for significance testing of PLS and OPLS (R) models. *Journal of Chemometrics.* 2008;22(11-12):594-600.

14. Wiklund S, Johansson E, Sjostrom L, et al. Visualization of GC/TOF-MS-based metabolomics data for identification of biochemically interesting compounds using OPLS class models. *Analytical chemistry.* 2008;80(1):115-122.

15. Xie C, Mao X, Huang J, et al. KOBAS 2.0: a web server for annotation and identification of enriched pathways and diseases. *Nucleic acids research.* 2011;39(Web Server issue):W316-322.

16. Kanehisa M, Goto S, Sato Y, Furumichi M, Tanabe M. KEGG for integration and interpretation of large-scale molecular data sets. *Nucleic acids research.* 2012;40(Database issue):D109-114.

17. Hoffmann RF, Zarrintan S, Brandenburg SM, et al. Prolonged cigarette smoke exposure alters mitochondrial structure and function in airway epithelial cells. *Respiratory research.* 2013;14:97.

18. Kirkham PA, Barnes PJ. Oxidative stress in COPD. *Chest.* 2013;144(1):266-273.

19. Repine JE, Bast A, Lankhorst I. Oxidative stress in chronic obstructive pulmonary disease. Oxidative Stress Study Group. *American journal of respiratory and critical care medicine.* 1997;156(2 Pt 1):341-357.

20. Rahman I, MacNee W. Lung glutathione and oxidative stress: implications in cigarette smoke-induced airway disease. *The American journal of physiology.* 1999;277(6 Pt 1):L1067-1088.

21. Hakim IA, Harris R, Garland L, Cordova CA, Mikhael DM, Sherry Chow HH. Gender difference in systemic oxidative stress and antioxidant capacity in current and former heavy smokers. *Cancer Epidemiol Biomarkers Prev.* 2012;21(12):2193-2200.

22. Prescott E, Bjerg AM, Andersen PK, Lange P, Vestbo J. Gender difference in smoking effects on lung function and risk of hospitalization for COPD: results from a Danish longitudinal population study. *Eur. Respir. J.* 1997;10(4):822-827.

23. Downs SH, Brandli O, Zellweger JP, et al. Accelerated decline in lung function in smoking women with airway obstruction: SAPALDIA 2 cohort study. *Respir. Res.* 2005;6:45.

24. Tam A, Churg A, Wright JL, et al. Sex Differences in Airway Remodeling in a Mouse Model of Chronic Obstructive Pulmonary Disease. *American journal of respiratory and critical care medicine.* 2016;193(8):825-834.

25. Naz S, Kolmert J, Yang M, et al. Metabolomics analysis identifies gender-associated metabotypes of oxidative stress and the autotaxin-lysoPA axis in COPD. *The European respiratory journal.* 2017;In press.

# Supplemental Figures


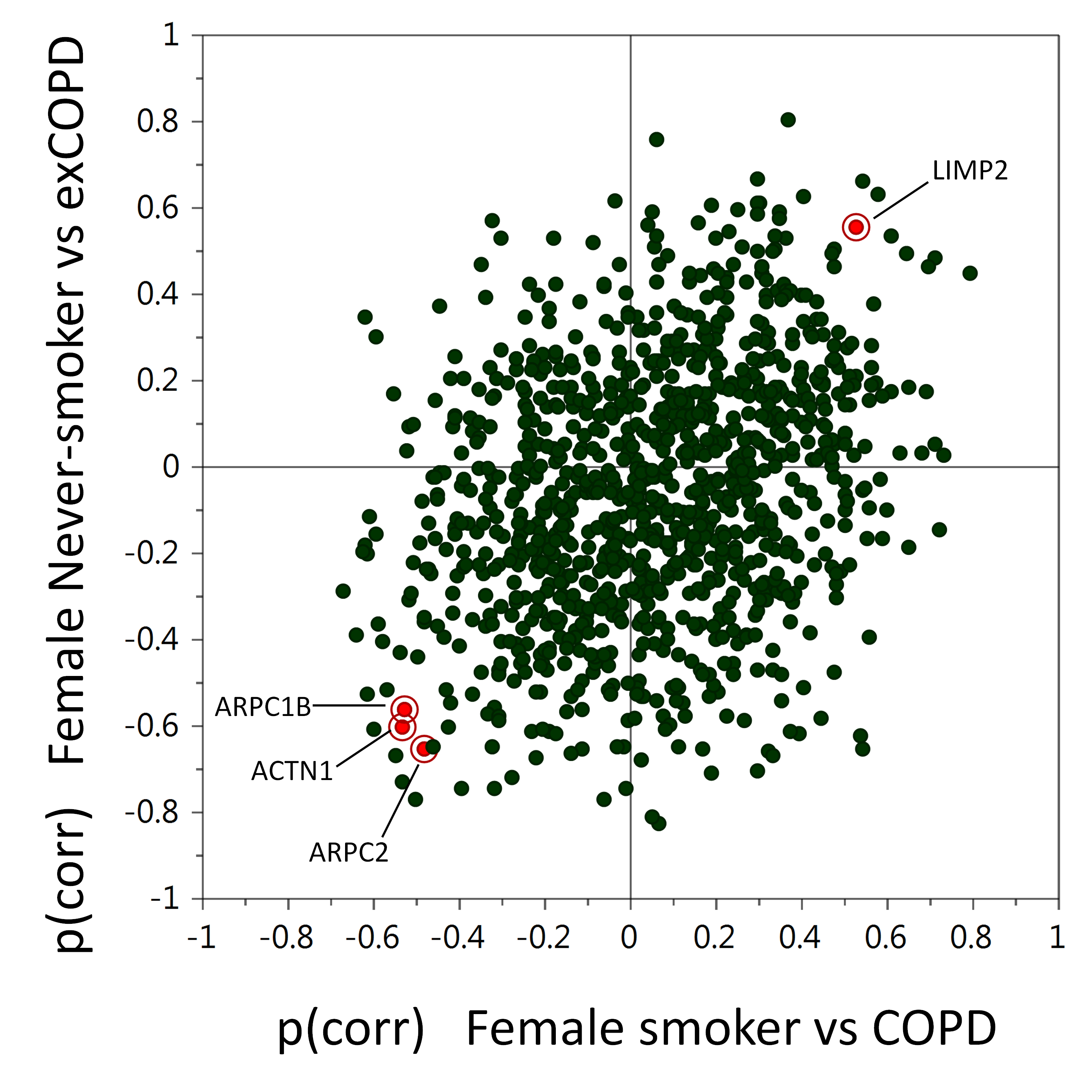


**Figure S1**. Analysis of Share and Unique Structure (SUS) between OPLS-DA models of female Smoker vs COPD (x-axis) and Never-smoker vs ex-smoker with COPD (exCOPD) (y-axis). These anlysis showed that 3 proteins (ARPC2, ACTN1 and ARPC1B) decreased in FcγR-mediated phagocytosis in both female current-smoker COPD patients as well as female ex-smoker COPD patients, indicating that dysregulation of FcγR-mediated phagocytosis partly is independent of current smoking status. In addition, the protein LIMP2 in the lysosomal pathway was up-regulated in female COPD patients both in the smoking and non-smoking polulations. (Never-smoker, n=9; exCOPD, n=5).


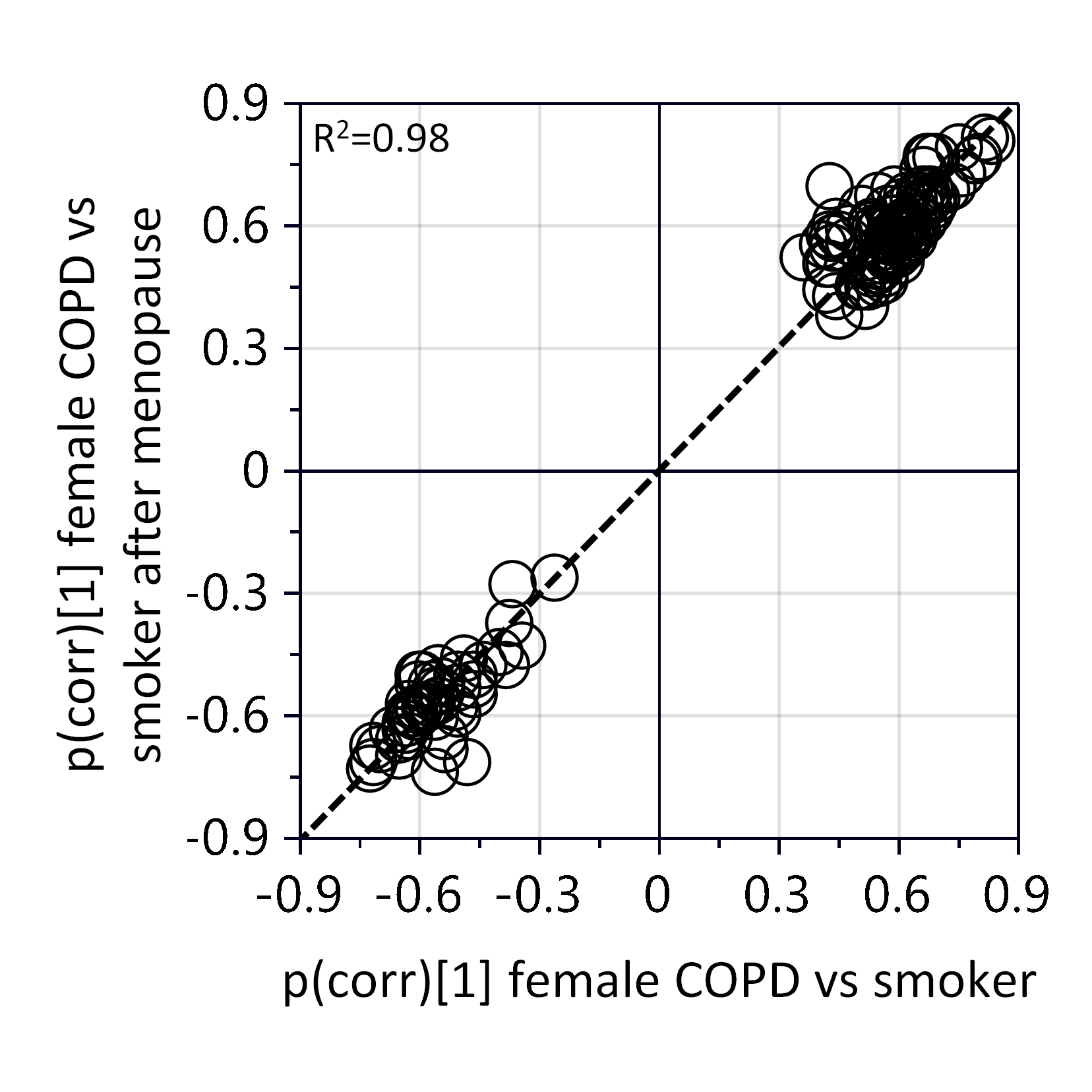


Figure S2. Multivariate sensitivity analysis of the impact of menopausal status on proteomic profiling in female COPD patients. SUS analysis showed that the correlation between models with and without pre-menopausal women was very high (R^2^=0.98), indicating that the observed expression proteins were not dependent on menopausal status. The model statistics of the original model (x-axis) is shown in Figure 3 b, and the model excluding pre-menopausal subjects (y-axis) was very similar (R^2^=0.86, Q^2^=0.80, p[CV-ANOVA]=2.4×10^-6^; Y-intercepts of permutation test: R^2^=0.42, Q^2^=-0.42).


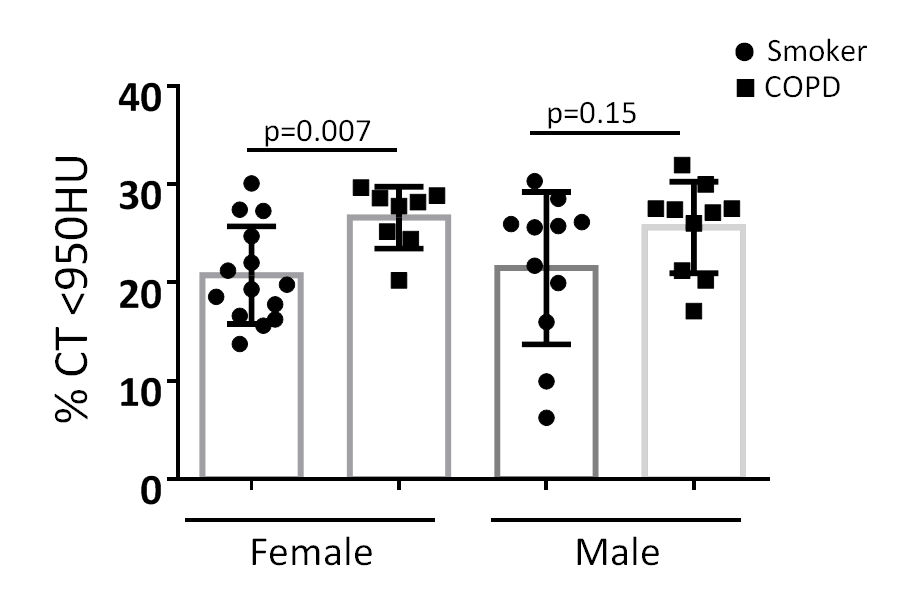


Figure S3. The percentage of CT attenuation values <-950 HU in the Smoker and COPD groups, stratified by gender. The proportion of attenuation values <-950 HU increased in female current-smoker COPD patients compared to the Smoker group (p=0.007). In contrast, the difference in the proportion of attenuation values <-950 HU between male current-smoker COPD patients and the Smoker group was not significant (p=0.15).
